# Electronic structure and thermal conductance of the MASnI_3_/Bi_2_Te_3_ interface: a first-principles study

**DOI:** 10.1038/s41598-021-04234-3

**Published:** 2022-01-07

**Authors:** Masayuki Morimoto, Shoya Kawano, Shotaro Miyamoto, Koji Miyazaki, Shuzi Hayase, Satoshi Iikubo

**Affiliations:** 1 Department of Life and Systems Engineering, Kyushu Institute of Technology, Kitakyushu Science and Research Park, Fukuoka, 808-0196 Japan; 2 Department of Mechanical and Control Engineering, Kyushu Institute of Technology, 1-1 Sensui-cho, Tobata-ku, Kitakyushu, 804-8550 Japan; 3 Info-Powered Energy System Research Center (I-PERC), The University of Electro-Communications, 1-5-1 Chofugaoka, Chofu, Tokyo, 182-8585 Japan; 4 Department of Advanced Materials Science and Engineering, Faculty of Engineering Sciences, Kyushu University, Kasuga, Fukuoka, 816-8580 Japan

**Keywords:** Thermoelectrics, Atomistic models

## Abstract

To develop high-performance thermoelectric devices that can be created using printing technology, the interface of a composite material composed of MASnI_3_ and Bi_2_Te_3_, which individually show excellent thermoelectric performance, was studied based on first-principles calculations. The structural stability, electronic state, and interfacial thermal conductance of the interface between Bi_2_Te_3_ and MASnI_3_ were evaluated. Among the interface structure models, we found stable interface structures and revealed their specific electronic states. Around the Fermi energy, the interface structures with Te^II^ and Bi terminations exhibited interface levels attributed to the overlapping electron densities for Bi_2_Te_3_ and MASnI_3_ at the interface. Calculation of the interfacial thermal conductance using the diffuse mismatch model suggested that construction of the interface between Bi_2_Te_3_ and MASnI_3_ could reduce the thermal conductivity. The obtained value was similar to the experimental value for the inorganic/organic interface.

## Introduction

Printed electronics are attractive next-generation technologies that make full use of printing technology and are used to manufacture various electronic devices, such as organic EL TVs, electronic paper, and solar cells. In addition to making the devices thinner and lighter and increasing the area, printed electronics also save resources, allow for reduced process temperatures, and are more environmentally friendly. In the field of printed electronics, the thermoelectric conversion demand has increased with the development of energy-saving technology, which has a crucial role in the achievement of energy harvesting^1–3^. Madan et al.^4^ and Navone et al.^5^ reported early composite printing of thermoelectric devices. Navone et al.^5^ also noted that a previous study^6^ attempted to achieve this in 1990 using screen printing. For thermoelectric devices, it is necessary to improve the energy conversion efficiency in addition to reducing costs. The performance of thermoelectric conversion materials is described as the figure of merit, *ZT* = *S*^2^*σT*/(*κ*_*el*_ + *κ*_*lat*_), which is proportional to the square of the Seebeck coefficient (*S*), electrical conductivity (*σ*), and absolute temperature (*T*), and is inversely proportional to the thermal conductivity (*κ*)^7–9^. Many studies have been conducted on the doping of heterogeneous elements to modify the electronic structure directly; this technique is effective for preventing an increase in the Seebeck coefficient and/or the electrical conductivity^10–12^.

On the other hand, a thousand-fold reduction in thermal conductivity was reported with the use of organic/inorganic interfaces^13^. This broadened the outlook of the field because the previous strategy improved semiconductor characteristics by controlling impurities, but new characteristics arose in the obtained composites. To decrease the lattice thermal conductivity, many researchers have introduced crystal grain boundaries and nanostructures, which led to phonon scattering between crystal grains^14–19^. These techniques also affect improving the electrical conductivity and Seebeck coefficient, and their mechanisms are complex. As mentioned above, the interfacial thermal conductance has attracted attention for enhancing *ZT*, and this enhancement is attributed to the difference in lattice vibrations between heterogeneous materials^3,20,21^. Considering the fabrication of thin and flexible thermoelectric materials, hybridization of organic and inorganic materials is a critical technique for improving *ZT* owing to the formation of many interfaces^3,8,22–24^. Generally, to maintain the electric conduction ability, the organic materials used are conductive polymers such as poly (3,4-ethylenedioxythiophene) doped with poly (4-styrenesulfonate) (PEDOT:PSS)^25,26^. Lu et al. developed flexible PEDOT:PSS/Cu_2_Se nanocomposite films on nylon, and the resulting material exhibited a relatively high power factor of ~ 389.7 µW/(m·K^2^) at 418 K^27^. Kumar et al. fabricated a PEDOT:PSS/Te composite material, which reduced the thermal conductivity owing to the enhanced phonon–phonon scattering in the polymer matrix^28^. Many researchers have also studied the combination of Bi_2_Te_3_ and PEDOT:PSS^8,29–32^. Du et al. fabricated Bi_2_Te_3_ based alloy nanosheet/PEDOT:PSS composite films, which exhibited high electrical conductivity (1295.21 S/cm) relative to BI_2_Te_3_-based alloy bulk materials (850–1250 S/cm), and a power factor of ~ 32.26 µW/(m·K^2^) was obtained^30^. In a Te-Bi_2_Te_3_/PEDOT:PSS hybrid film synthesized through a solution-phase reaction at low temperature, a power factor of 60.05 µW/(m·K^2^) with a Seebeck coefficient of 93.63 µV/K and an electrical conductivity of 69.99 S/cm were reported by Bae et al^31^. Based on these results, it can be concluded that the electronic properties of the interface between the organic and inorganic materials play a critical role in improving the *ZT* of organic–inorganic hybrid materials.

Here, we focus on halide perovskites instead of PEDOT:PSS to fabricate a printable thermoelectric material. The thermoelectric properties of inorganic halide perovskite (CsSnI_3_) have previously demonstrated relatively high values as a printable thermoelectric material (ZT > 0.1 at room temperature)^33,34^. Organic–inorganic hybrid perovskites, ABX_3_ (A: methylammonium cation (CH_3_NH_3_^+^), B: lead or tin, X: iodide) have been investigated as candidate thermoelectric materials and are well known in the field of thin-film solar cells^35,36^. Regarding the thermoelectric properties of organic–inorganic perovskites, Pisoni et al. reported that CH_3_NH_3_PbI_3_ exhibited an ultra-low thermal conductivity of 0.3–0.5 W/(m·K) at room temperature due to the slowly rotating CH_3_NH_3_^+^ cations within the crystal structure^37^. Theoretical studies also predicted that CH_3_NH_3_PbI_3_ would have a low thermal conductivity of ~ 1 W/(m·K) compared with other perovskites such as CsPbI_3_, CH_3_NH_3_Br_3_, and CH_3_NH_3_PbCl_3_^38–40^. On the other hand, CH_3_NH_3_SnI_3_ (MASnI_3_) is expected to exhibit low thermal conductivity compared with CH_3_NH_3_PbI_3_, with improved thermal properties obtained through chemical doping^41^. The advantage of perovskite compounds such as MASnI_3_ over the organic materials PEDOT: PSS is that they have a variety of constituent elements, which enable the system elemental substitution. It is possible to change the energy level near the Fermi level, and it is expected that the electric conductivity and Seebeck coefficient will be improved. Such electronic state control can be performed more easily with perovskite than with PEDOT:PSS.

In this study, we aimed to understand the interface structure of hybrid materials composed of Bi_2_Te_3_ and organic–inorganic perovskite (MASnI_3_) to improve the thermoelectric conversion properties. We previously reported the structural stability and electronic properties of different Bi_2_Te_3_ (001) termination surfaces based on first-principles calculations^42^. Based on the results, we prepared three structures with different Bi_2_Te_3_ termination structures and explored statically stable structures through structural optimization. Additionally, we calculated the electronic states and distribution of the charge density near the Fermi energy. The calculation of the diffuse mismatch model (DMM)^43,44^ obtained from the results of phonon dispersion in Bi_2_Te_3_ and MASnI_3_ confirmed a decrease in the interfacial thermal conductance at the interface.

## Computational methods

### Density functional theory calculations for Bi_2_Te_3_/MASnI_3_ interfaces

**Figure 1:**
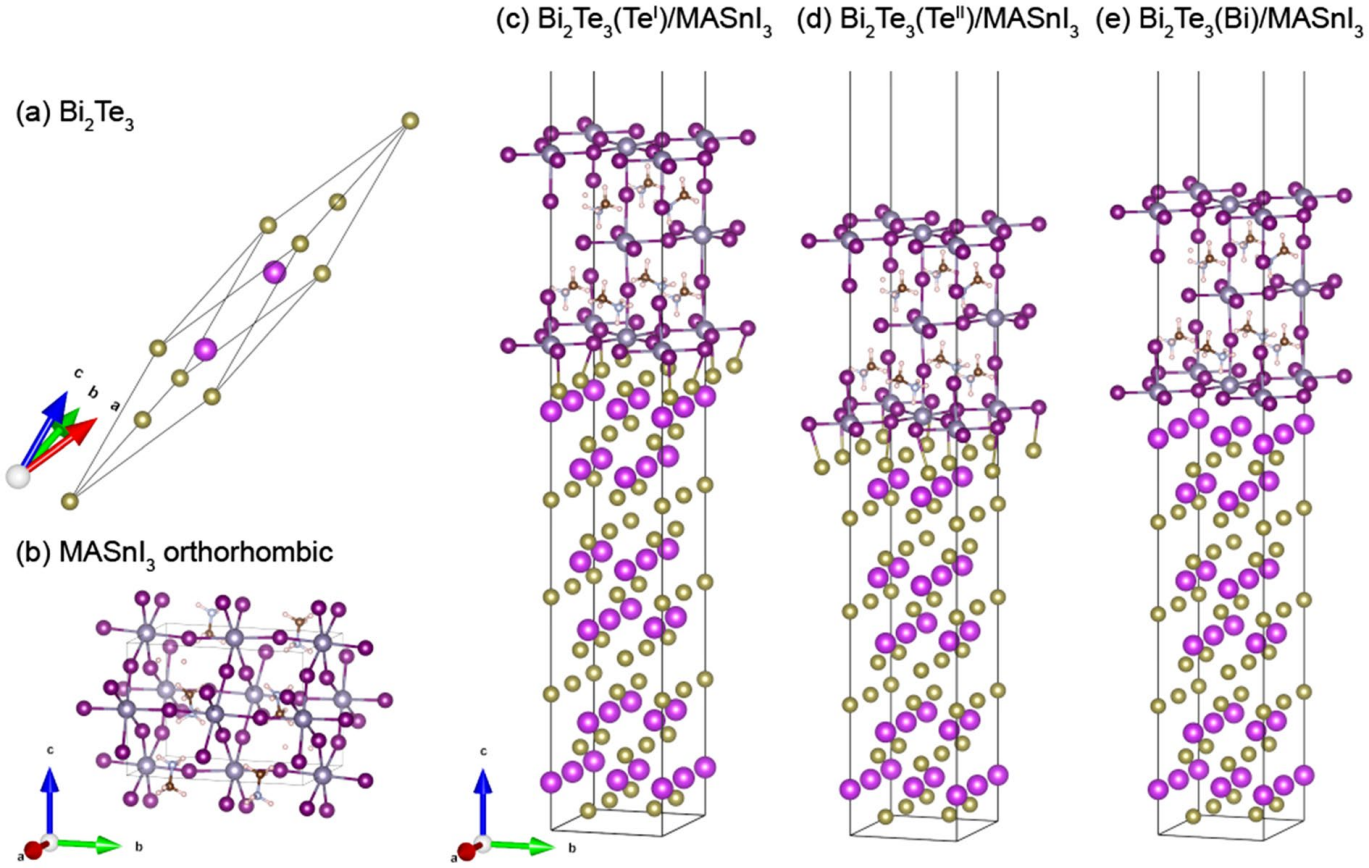
Structural models of (**a**) Bi_2_Te_3_ and (**b**) orthorhombic MASnI_3_ used for the DMM calculation; calculated interface structures of (**c**) Bi_2_Te_3_(Te^I^)/MASnI_3_, (**d**) Bi_2_Te_3_(Te^II^)/MASnI_3_, and (**e**) Bi_2_Te_3_(Bi)/MASnI_3_. The interface crystal plane is (001). The (001) crystal plane was used for both Bi_2_Te_3_ and MASnI_3_, with an ~ 15 Å vacuum layer inserted.

To create the interface structure, the crystal structure of Bi_2_Te_3_ was transformed from a rhombohedral lattice to an orthorhombic lattice, and the lattice parameter of MASnI_3_ was reduced to fit the lattice parameter of Bi_2_Te_3_. The interface models consisted of orthorhombic Bi_2_Te_3_ (001) and tetragonal MASnI_3_ (001), and a vacuum layer of ~ 15 Å was inserted. For simplicity, the termination structure of MASnI_3_ was fixed as SnI_2_ at the interface. For the structure of Bi_2_Te_3_ in contact with MASnI_3_, three termination structures were considered: Te^I^, Te^II^, and Bi terminations, which are relatively stable surface structures that were described in our previous study^42^ (Fig. 1c–e). The Vienna ab-initio simulation package (VASP)^45,46^ with the projector-augmented wave method^47,48^ was used for the first-principles calculations. For the exchange–correlation function, the generalized gradient approximation and Perdew–Burk–Ernzerhof function were used^49^. The cutoff energy was set at 520 eV, and structural optimization was performed using the Gaussian smearing method with a sigma value of 0.1 eV. The K-points were set at 5 × 6 × 1, and the convergence value for the structural optimization was set to 10^−3^ eV. The Blöchl-corrected tetrahedron method was used for accurate calculation, and its convergence value was set at 10^−4^ eV. To perform more accurate band structure, density of states (DOS), and charge distribution, we considered the spin–orbit coupling (SOC).

### Calculation of thermal conductance using DMM

For the thermal conductance calculation, phonon calculation of the interface structure between Bi_2_Te_3_ and MASnI_3_ is the most direct calculation method. However, for an interface structure, the number of atomic displacement patterns are required to obtain the highly accurate atomic force, and it is impossible to calculate by the first-principles calculation. Therefore, in this paper, we used DMM^44^, which is often used as a simple method for evaluating interfacial thermal conductance.

The interfacial thermal conductance (thermal boundary conductance) obtained by the DMM is defined as the ratio of the heat current density to the temperature differential. To estimate the thermal boundary conductance for hybrid materials A/B, Reddy et al. defined the thermal boundary conductance, *G*, as follows:

1 $$ G = \frac{1}{{2\left( {2\pi } \right)^{3} }}\mathop \sum \limits_{i} \mathop \int \limits_{{\varvec{k}}} \frac{1}{{k_{B} T^{2} }}\alpha_{A \to B} \left( {{\varvec{k}},i} \right) \times \left( {\hbar \omega \left( {{\varvec{k}},i} \right)} \right)^{2} \left| {{\varvec{V}}\left( {{\varvec{k}},i} \right).{\varvec{n}}} \right|\frac{{{\text{exp}}\left( {\frac{{\hbar \omega \left( {{\varvec{k}},i} \right)}}{{k_{B} T}}} \right)}}{{\left[ {{\text{exp}}\left( {\frac{{\hbar \omega \left( {{\varvec{k}},i} \right)}}{{k_{B} T}}} \right) - 1} \right]^{2} }}d\user2{k,} $$

where $\alpha_{A \to B} \left( {{\varvec{k}},i} \right)$ is the transmission probability of A to B, $\omega \left( {{\varvec{k}},i} \right)$ is the phonon frequency corresponding to wave vector ***k*** and phonon mode *I*, and $\left| {{\varvec{V}}\left( {{\varvec{k}},i} \right).{\varvec{n}}} \right|$ is the group velocity along the unit vector ***n*** to the interface of A to B. Calculations of the transmission probability of A to B and the phonon frequency and group velocity of A and B obtained from phonon dispersion are required. Here, the transmission probability is calculated from the group velocities of A and B as follows:

2 $$ \alpha_{A \to B} \left( {\omega^{\prime } } \right) = \frac{{\Delta K_{B} \left[ {\mathop \sum \nolimits_{{j,{\varvec{k}}}} \left| {{\varvec{V}}\left( {{\varvec{k}},j} \right).{\varvec{n}}} \right|} \right]\delta_{{\omega \left( {{\varvec{k}},j} \right),\omega^{\prime } }} }}{{\Delta K_{A} \left[ {\mathop \sum \nolimits_{{i,{\varvec{k}}}} \left| {{\varvec{V}}\left( {{\varvec{k}},i} \right).{\varvec{n}}} \right|} \right]\delta_{{\omega \left( {{\varvec{k}},i} \right),\omega^{\prime } }} + \Delta K_{B} \left[ {\mathop \sum \nolimits_{{j,{\varvec{k}}}} \left| {{\varvec{V}}\left( {{\varvec{k}},j} \right).{\varvec{n}}} \right|} \right]\delta_{{\omega \left( {{\varvec{k}},j} \right),\omega^{\prime } }} }}, $$

where $\Delta K_{A}$ and $\Delta K_{B}$ are the discretized cells of the Brillouin zones of A and B, respectively, and $\delta_{{\omega \left( {{\varvec{k}},i} \right),\omega^{{^{\prime } }} }}$ is the Kronecker delta function. Therefore, to evaluate the thermal boundary conductance with DMM, only the phonon dispersions of A and B are required. The calculated thermal conductance will be severely underestimated (by a factor of 1/2) when the transmission probability between similar materials is calculated using the DMM. Therefore, the maximum transmission model (MTM) was employed to evaluate the extreme upper limit of the thermal conductance if needed^50^.

The phonon dispersions of Bi_2_Te_3_ and MASnI_3_ were evaluated using first-principles phonon calculations, and the group velocity was calculated from the results. To calculate the phonon dispersion, we used the finite displacement method with a displacement distance of 0.01 Å. The supercell sizes of Bi_2_Te_3_ and MASnI_3_ were 2 × 2 × 2 for the rhombohedral cells and 1 × 1 × 1 for the orthorhombic cells, respectively (Fig. 1a,b). We note that the longer lattice parameter of the orthorhombic cell of MASnI_3_ is along the b-axis, and the a- and b-axes are rotated 45° relative to the cubic perovskite phase. To estimate the force due to the introduction of displacements, we used the VASP code with the following parameters: a plane wave energy cutoff of 400 eV, a convergence value for the electronic self-consistency loop of 10^−8^ eV, Γ-point centered k-mesh limited to 0.1 Å^−1^, and the Gaussian smearing method with a smearing width of 0.05 eV. In the phonon calculation, SOC does not significantly affect the phonon dispersion relation, therefore, SOC is not considered. We used the phonopy code^51^ to create the displacement using the finite displacement method, and the ALAMODE^52^ code for the phonon properties calculation. To obtain the phonon density of states (DOS) and group velocity for both structures, the reciprocal space was sampled using 10 × 10 × 10 meshes.

## Results and discussion

### Optimized interface structure

We constructed three interface structures with different Bi_2_Te_3_ termination structures: Bi_2_Te_3_(Te^I^)/MASnI_3_, Bi_2_Te_3_(Te^II^)/MASnI_3_, and Bi_2_Te_3_(Bi)/MASnI_3_ (Fig. 1). The crystal plane in contact with each structure in the interface was determined from the lowest lattice deformation ratio of various combinations of crystal planes; the selected structure of MASnI_3_ was tetragonal, which was stable at room temperature. In the creation of the interface models, the lattice distance of MASnI_3_ was reduced to fit that of Bi_2_Te_3_.

Table 1 lists the lattice parameters of various interface models after structural optimization. The termination structure of Bi_2_Te_3_ affected the lattice constant of the interface model; the Te^II^ termination exhibited the lowest values for the a- and b-axes compared with the Te^I^ and Bi termination structures (Table 1). This result also led to a decrease in the lattice deformation ratio in the Bi_2_Te_3_(Te^II^)/MASnI_3_ structure, as indicated in Table 2. The lattice deformation ratio was calculated using the following equation: deformation (%) = (*d*_2_/*d*_1_ – 1) × 100, where *d*_2_ and *d*_1_ represent the lattice distance of the transformed or optimized structure and the lattice distance of the bulk structure, respectively. On the other hand, the Bi_2_Te_3_(Bi)/MASnI_3_ structure exhibited a high lattice deformation ratio among the three interface models; the lattice of Bi_2_Te_3_ in the interface structure was particularly expanded. A low lattice deformation ratio is expected in the case of easy formation and relatively high stability of the interface structure experimentally.

**Table 1:** Lattice parameters of various interfaces after structural optimization.

	a (Å)	b (Å)	c (Å)	α (°)	β (°)	γ (°)
Bi_2_Te_3_(Te^I^)/MASnI_3_	8.9175	7.9201	63.756	88.38	88.82	90.02
Bi_2_Te_3_(Te^II^)/MASnI_3_	8.8533	7.8737	62.240	91.08	90.13	89.80
Bi_2_Te_3_(Bi)/MASnI_3_	8.9404	7.9758	60.173	90.58	90.62	89.49

**Table 2:** Lattice deformation ratios of various interfaces after structural optimization.

	Lattice deformation (Bi_2_Te_3_)^a^		Lattice deformation (MASnI_3_)^b^	
	a-axis (%)	b-axis (%)	a-axis (%)	b-axis (%)
Bi_2_Te_3_(Te^I^)/MASnI_3_	1.644	4.243	1.071	− 10.23
Bi_2_Te_3_(Te^II^)/MASnI_3_	0.913	3.631	0.343	− 10.76
Bi_2_Te_3_(Bi)/MASnI_3_	1.907	4.975	1.331	− 9.602

^a^This value represents the lattice deformation ratio of the Bi_2_Te_3_ structure in the interface model based on the bulk orthorhombic Bi_2_Te_3_ bulk structure before optimization.

^b^This value represents the lattice deformation ratio of the MASnI_3_ structure in the interface model based on the tetragonal MASnI_3_ bulk structure before optimization.

After structural optimization, the atoms in the interface moved strongly with an incomplete structure for Bi_2_Te_3_ with the Te^II^ and Bi termination structures, and this phenomenon was prominent for the Bi termination. This result suggests that Bi and Sn atoms can move easily into each structure, and the Te^II^ and Bi termination structures form an interaction between Bi_2_Te_3_ and MASnI_3_ compared with the Te^I^ termination. The relationship between the reconstruction of atoms in the structural optimization, lattice parameters, and lattice deformation ratio was not observed.

**Figure 2:**
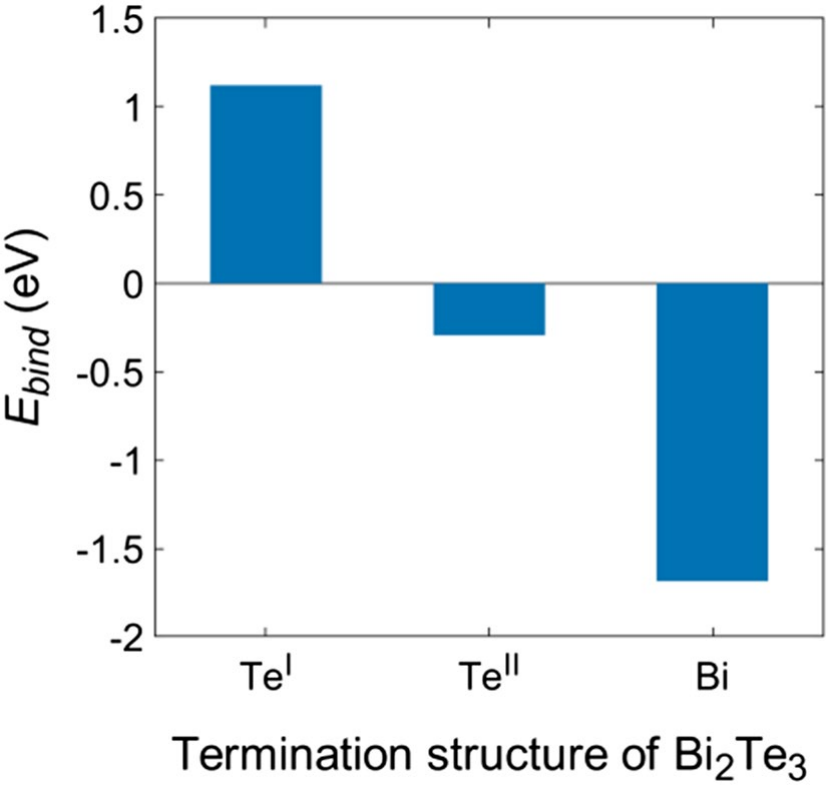
Binding energies of various Bi_2_Te_3_/MASnI_3_ interfaces with different Bi_2_Te_3_ termination structures.

To evaluate the interface stability between Bi_2_Te_3_ and MASnI_3_, we calculated the binding energy using the following equation: *E*_*bind*_ = *E*_*total*_ – *E*_*p*_ – *E*_*b*_, where *E*_*total*_, *E*_*p*_, and *E*_*b*_ denote the energies of Bi_2_Te_3_/MASnI_3_, the MASnI_3_ (001) surface, and the Bi_2_Te_3_ (001) surface, respectively (Fig. 2). *E*_*p*_, and *E*_*b*_ means reference energies. For these (001) surface structures, we used the lattice constant of the ground state structure. A positive binding energy value indicates low stability of the interface structure, which makes formation of the interface difficult. Hence, Bi_2_Te_3_(Te^I^)/MASnI_3_ is the most unstable interface structure; in contrast, Bi_2_Te_3_(Bi)/MASnI_3_ is the most stable interface structure, with a binding energy of − 1.7 eV. Similar to the Bi termination model, Bi_2_Te_3_(Te^II^)/MASnI_3_ exhibited a negative binding energy of − 0.3 eV. Note that the corresponding interfacial energy, which is represented by energy/area, are 1.59 eV/nm^2^ for Bi_2_Te_3_(Te^I^)/MASnI, − 0.42 eV/nm^2^ for Bi_2_Te_3_(Te^II^)/MASnI_3_, and − 2.36 eV/nm^2^ for Bi_2_Te_3_(Bi)/MASnI_3_. The optimized structures suggest that the stability of the interface structure is attributable to the reconstruction of atoms in the interface.

### Electronic properties of Bi_2_Te_3_/MASnI_3_ interfaces

**Figure 3:**
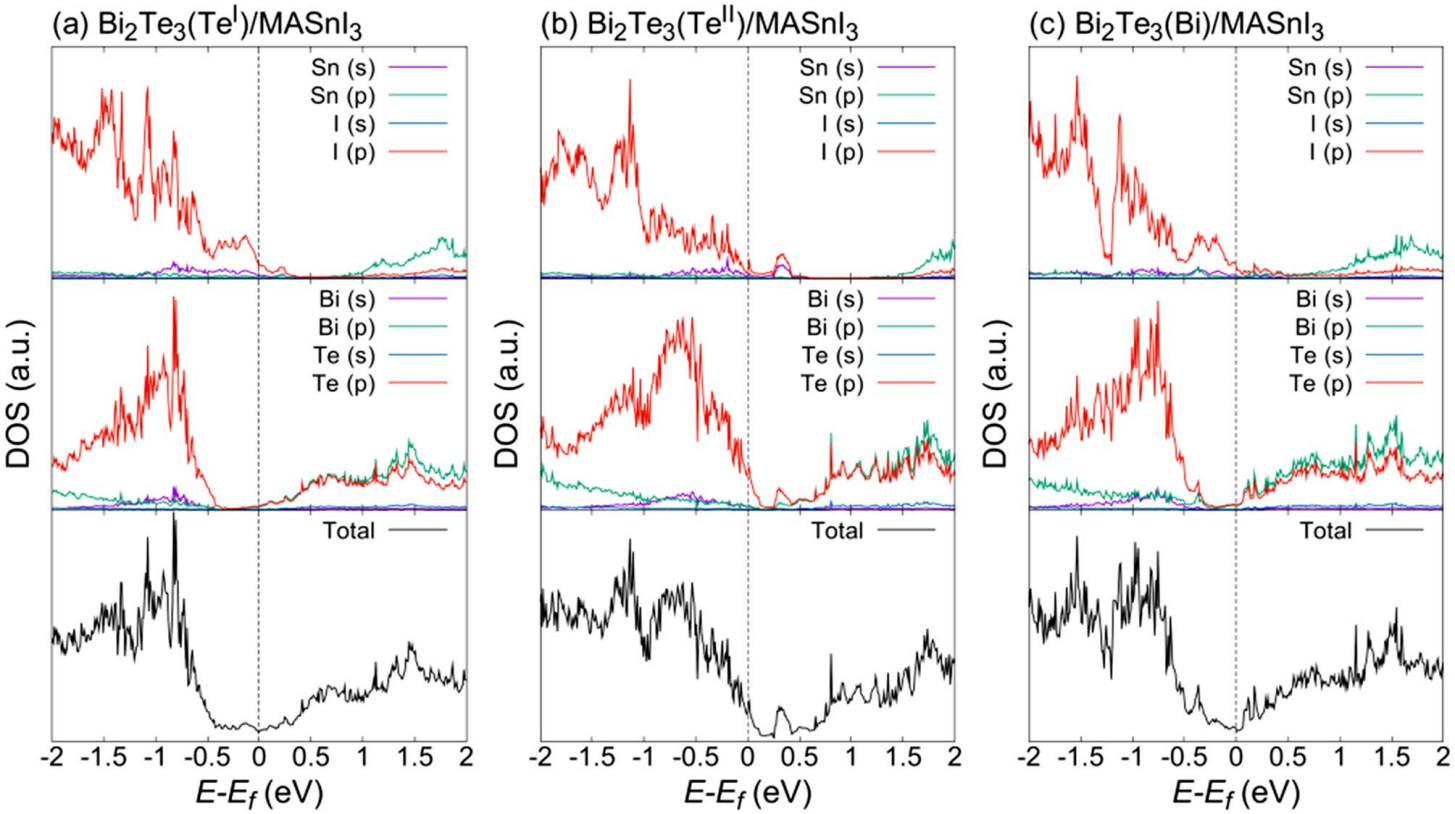
Total and partial DOS around the Fermi energy of (**a**) Bi_2_Te_3_(Te^I^)/MASnI_3_, (**b**) Bi_2_Te_3_(Te^II^)/MASnI_3_, and (**c**) Bi_2_Te_3_(Bi)/MASnI_3_.

The total and partial DOS around the Fermi energy for various Bi_2_Te_3_/MASnI_3_ interfaces are shown in Fig. 3 (corresponding band structures are also shown in Fig. S1). The valence band of each interface structure consists of Sn s-, I p-, Bi s-, Bi p-, and Te p-orbitals, and the conduction band consists of Sn p-, I p-, Bi p-, and Te p-orbitals. In the interface structures, the shapes of the partial DOS of Bi_2_Te_3_ in each termination structure were similar to that of the bulk structure. However, the energy levels of the DOS changed with the termination structure in Bi_2_Te_3_, which is attributed to the difference in the ratio of Bi and Te atoms. The partial DOS of MASnI_3_ in each interface structure exhibited different electronic states from the bulk structure over a range of − 0.5 eV to 0 eV; these states are attributed to the I p-orbital in MASnI_3_ in contact with the vacuum layer. In the DOS around the Fermi energy in the Te^II^ and Bi termination structures (Fig. 3b,c), the additional electronic state appeared at similar energy levels for both Bi_2_Te_3_ and MASnI_3,_ indicating that the additional electronic state includes the contributions of both Bi_2_Te_3_ and MASnI_3_.

**Figure 4:**
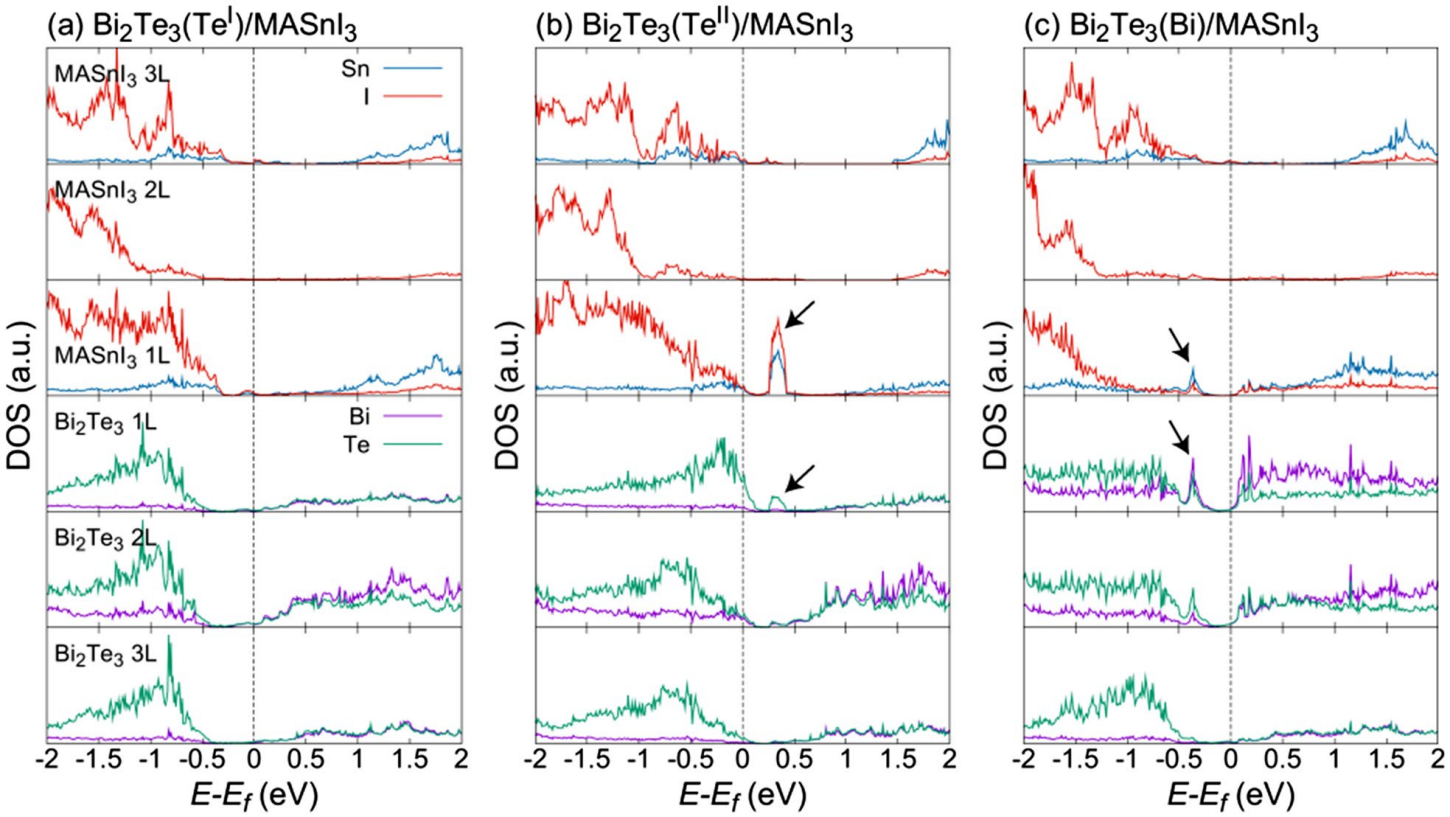
Decomposed DOS of each layer near interface for (**a**) Bi_2_Te_3_(Te^I^)/MASnI_3_; (**b**) Bi_2_Te_3_(Te^II^)/MASnI_3_; and (**c**) Bi_2_Te_3_(Bi)/MASnI_3_. The DOS of Bi, Te, Sn, and I consist of the sum of the s- and p-orbitals. The atoms included in each layer are shown in Fig. 5. The arrows indicate interface levels formed from Bi_2_Te_3_ and MASnI_3_.

**Figure 5:**
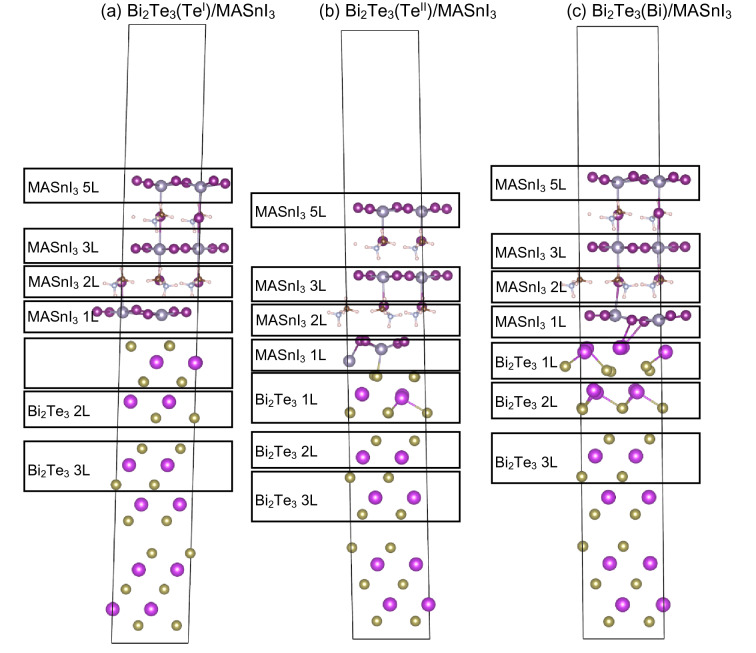
Decomposition method for each layer in the (**a**) Bi_2_Te_3_(Te^I^)/MASnI_3_, (**b**) Bi_2_Te_3_(Te^II^)/MASnI_3_, and (**c**) Bi_2_Te_3_(Bi)/MASnI_3_ interface structures and the atoms included in each layer.

To investigate the additional electronic state, the decomposed DOS for each layer near the interface is shown in Fig. 4. The atoms included in the decomposed layer are shown in Fig. 5. The DOS for Bi, Te, Sn, and I consist of the s- and p-orbitals. The DOS for the middle layer of MASnI_3_ (MASnI_3_-3L) exhibited a similar shape despite the different interface structures. However, the shapes of the DOS for Sn and Bi changed significantly near the interface, and they exhibited a different electronic state with the variation in Bi_2_Te_3_ termination. In particular, on the Bi_2_Te_3_(Bi)/MASnI_3_ interface, the conduction band of MASnI_3_-1L moved to near the Fermi energy. This result is attributed to the large change in the atomic positions of Sn and I at the interface. On the other hand, the shape of the DOS for Bi_2_Te_3_ depended on its termination structure; in particular, it changed significantly in the layer in contact with the interface. This phenomenon originates from the different ratios of Bi and Te atoms in the incomplete Bi_2_Te_3_ structure. The Bi_2_Te_3_(Bi)/MASnI_3_ interface also had the potential to be affected by the movement of Bi atoms.

Focusing on the first layers from the interface of Bi_2_Te_3_ and MASnI_3_, the additional electronic state is observed at the same energy level in the layer near the interface between Bi_2_Te_3_ and MASnI_3_. Figure 4(b) and (c) shows additional interface levels, denoted by arrows, with an overlapping electronic density appeared in both structures around the Fermi energy in the Bi_2_Te_3_(Te^II^)/MASnI_3_ and Bi_2_Te_3_(Bi)/MASnI_3_ structures. These results also suggest that the incomplete structure of Bi_2_Te_3_, such as the Te^II^ and Bi terminations, plays an important role in the formation of interface states. The Te^I^ termination did not produce an overlap in the DOS between Bi_2_Te_3_ and MASnI_3_ at the interface.

**Figure 6:**
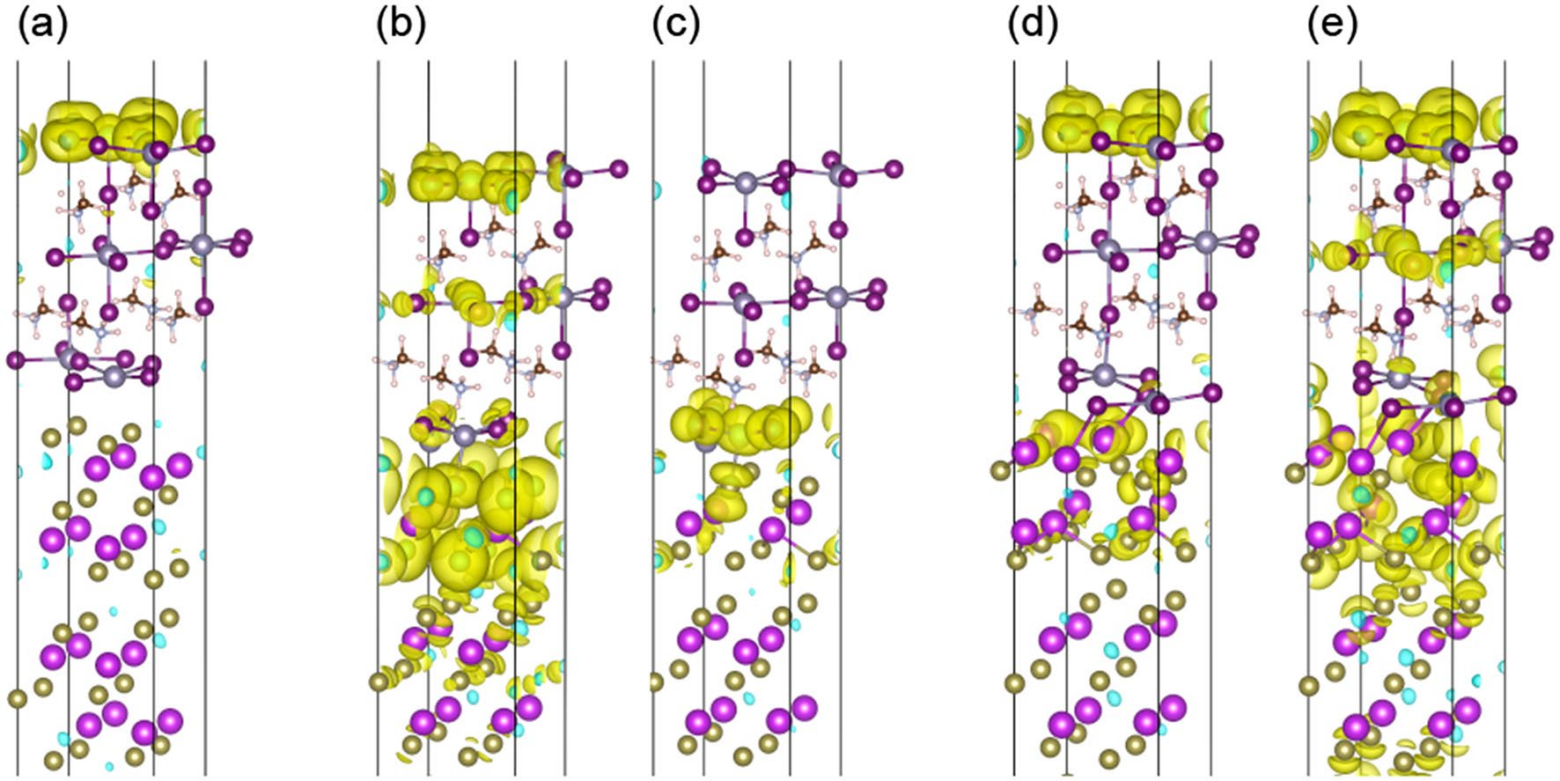
Visualization of charge distributions at selected energy levels for (**a**) Bi_2_Te_3_(Te^I^)/MASnI_3_; (**b**) and (**c**) Bi_2_Te_3_(Te^II^)/MASnI_3_; (**d**) and (**e**) Bi_2_Te_3_(Bi)/MASnI_3_. The indicated energy ranges are − 0.2 to 0.2 eV for (**a**), (**b**), and (**d**); 0.2–0.5 eV for (**c**); and − 0.5 to − 0.2 eV for (**e**).

The charge densities of each interface structure around the Fermi energy are shown in Fig. 6. Figure 6(a) shows the charge distribution in the interface structure with the Te^I^ termination, which is localized at the MASnI_3_ side, and is not observed at the interface between Bi_2_Te_3_ and MASnI_3_. This suggests a decreasing affinity of Bi_2_Te_3_ and MASnI_3_. On the other hand, the interface structure with the Te^II^ termination possesses a localized charge distribution at the near interface and an overlapping charge density between Sn and Te atoms in the energy range of 0.2 to 0.5 eV (Fig. 6c). Figure 6(d) and (e) show that the charge distribution of Bi_2_Te_3_(Bi)/MASnI_3_ is localized at the interface, similar to Bi_2_Te_3_(Te^II^)/MASnI_3_. In this case, the overlap of the charge density appears in the valence band of − 0.5 to − 0.2 eV. These results suggest that the overlapping of the charge distribution increases the affinity of the interface and assists in charge transfer between Bi_2_Te_3_ and MASnI_3_. For all interface structures, the charge density is localized at the surface of MASnI_3_ at − 0.2 eV to 0.2 eV. From the perspective of a thermoelectric material, the material requires a high electrical conductivity and Seebeck coefficient and a low thermal conductivity. The Seebeck coefficient is related to the shape of the DOS around the Fermi energy^53^, and the formation of interface levels may lead to a change in its value. Further work is needed to clarify the thermoelectric properties of the Bi_2_Te_3_/MASnI_3_ interface structure.

### Phonon properties of Bi_2_Te_3_ and MASnI_***3***_

**Figure 7:**
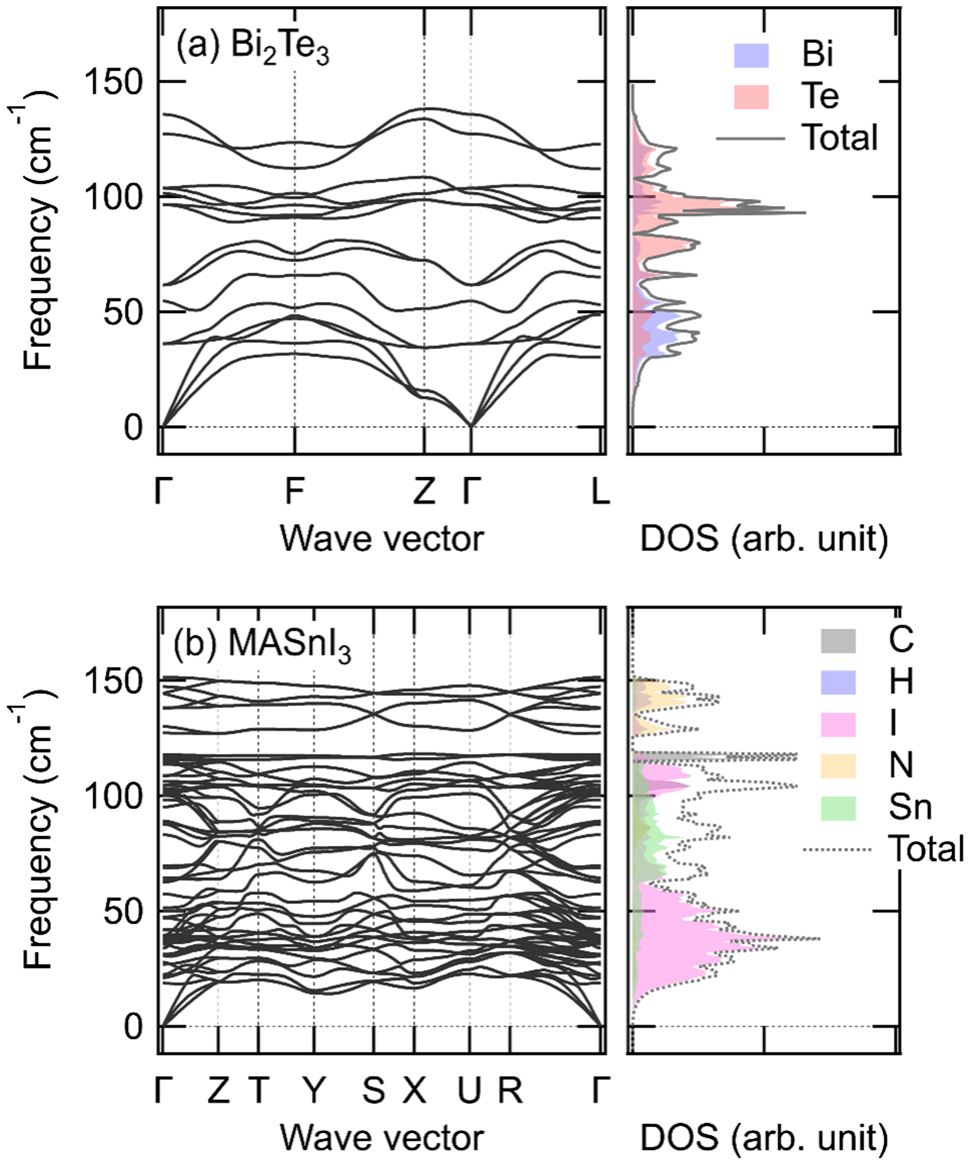
Phonon dispersions and atomic projected phonon DOS of (a) Bi_2_Te_3_ and (b) MASnI_3_.

Next, we estimated the interfacial thermal conductance of the Bi_2_Te_3_/MASnI_3_ interface. Figure 7 shows the phonon dispersions and atomic projected phonon DOS of (a) Bi_2_Te_3_ and (b) MASnI_3_. Bi_2_Te_3_ exhibited low energy phonon modes below 150 cm^−1^, and MASnI_3_ had low (f < 120 cm^−1^) and high (f > 120 cm^−1^) energy phonon modes. Because of the difference in atomic mass, the vibrations of Sn and I appeared at low energies, and the vibrations of C, N, and H appeared at high energies. Therefore, the phonon dispersion of MASnI_3_ showed a low-energy mode in the same range as Bi_2_Te_3_ until approximately 150 cm^−1^. Based on the phonon dispersion results for Bi_2_Te_3_ and MASnI_3_, these structures are dynamically stable at *T* = 0 owing to the lack of observation of the imaginary mode. Our calculated phonon dispersions for both structures are similar to those reported in previous studies for Bi_2_Te_3_^54^ and MAPbI_3_ (not MASnI_3_)^55,56^.

**Figure 8:**
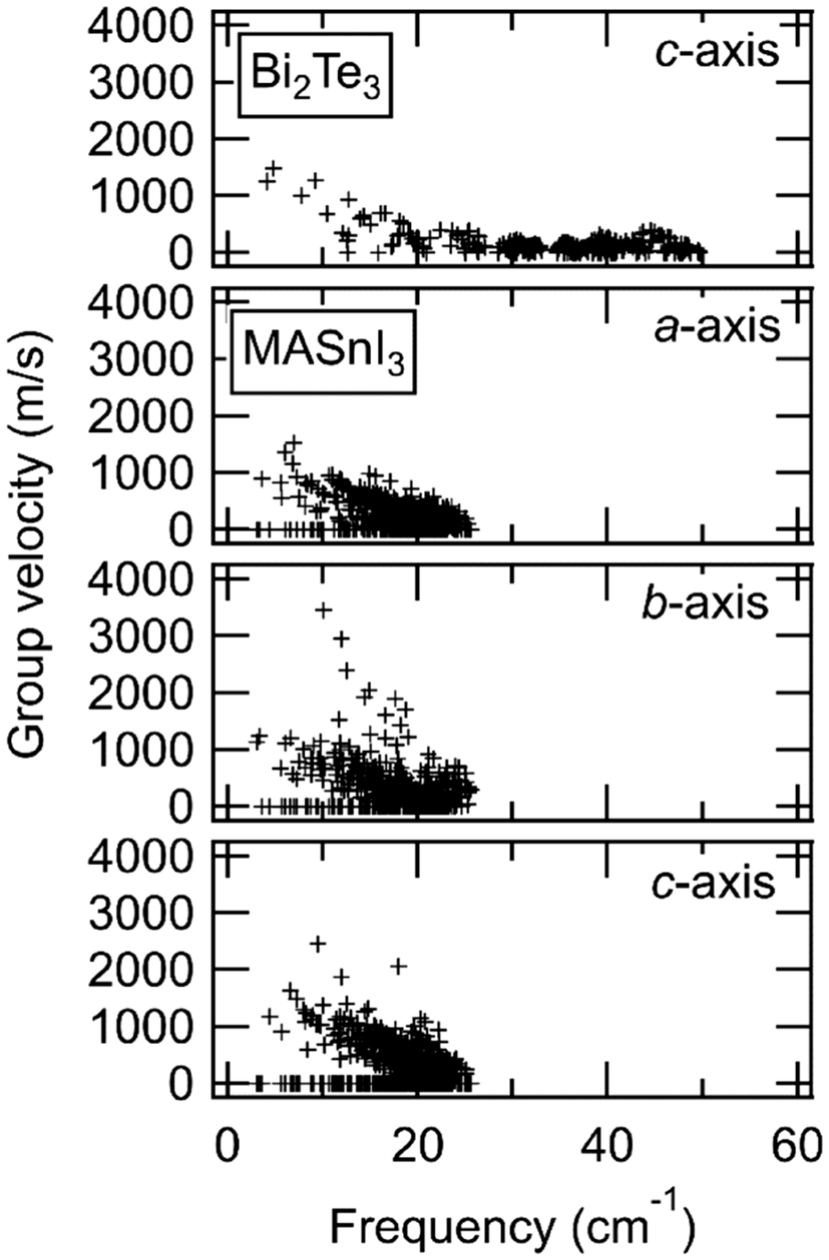
Group velocities of Bi_2_Te_3_ along the *c*-axis and MASnI_3_ along the *a*-axis, *b*-axis, and *c*-axis.

The group velocities of phonons are necessary for the evaluation of the interfacial thermal conductance using DMM, as shown in Eqs. (1) and (2). Figure 8 shows the absolute values of the calculated group velocities in the direction of the c-axis in Bi_2_Te_3_ and a-, b-, and c-axes in MASnI_3_. The group velocity (speed of sound) was estimated from the three low-energy phonon modes within the phonon dispersion; hence, it corresponds to a gradient of the phonon dispersion. In calculated results, Bi_2_Te_3_ exhibited a high group velocity at under 10 cm^−1^, whereas MASnI_3_ had a high group velocity above 10 cm^−1^. Moreover, we found that the distribution of the group velocity with respect to the frequency differed between Bi_2_Te_3_ and MASnI_3_. For MASnI_3_, the group velocity was not dependent on the direction of the crystal axis. It has been experimentally reported that Bi_2_Te_3_ exhibits a group velocity of 1750 m/s based on nuclear resonant inelastic scattering^57^, whereas MAPbI_3_ has exhibited group velocities of acoustic modes of 2400 or 1200 m/s based on neutron scattering^58^. Although there is a difference in the structures between our calculations (MASnI_3_) and the previous experiments (MAPbI_3_), our calculated values are approximately consistent with the experimental data.

### Interfacial thermal conductance between Bi_2_Te_3_ and MASnI_3_ at the interface

**Figure 9:**
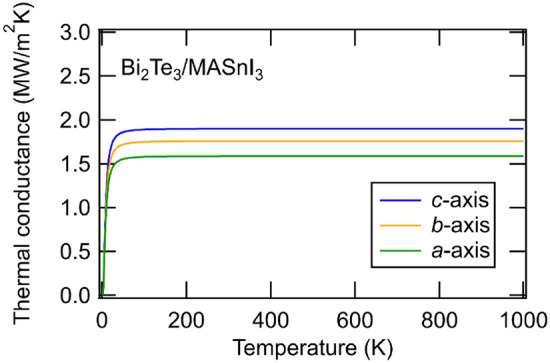
Interfacial thermal conductance of the Bi_2_Te_3_/MASnI_3_ interface along the a-axis, b-axis, and c-axis of MASnI_3_.

The interfacial thermal conductance of the Bi_2_Te_3_/MASnI_3_ interfaces was estimated by DMM using the group velocities of both structures. Figure 9 shows the interfacial thermal conductance of the Bi_2_Te_3_/MASnI_3_ interface; the combination of all axes of MASnI_3_ and the c-axis of Bi_2_Te_3_ was evaluated. The obtained values for the interfacial thermal conductance with different Bi_2_Te_3_/MASnI_3_ interfaces were 1.5–2.0 MW/m^2^K. This result indicates that the interfacial thermal conductance was not affected by the orientation of MASnI_3_ because the differences between different directions were small, as shown in the phonon dispersion curve (Fig. 7b). The calculated interfacial thermal conductance of Bi_2_Te_3_/MASnI_3_ was lower than the calculated value of the inorganic/inorganic interface^44^. The reason for this is explained as follows: calculated phonons in Bi_2_Te_3_ and MASnI_3_ are distributed in the low energy region. This is due to the fact that they have relatively heavy elements and complicated structures. When the phonon dispersion is distributed in the low energy region, the group velocity becomes small. The interfacial thermal conductance calculated by DMM depends on the group velocity and phonon frequency, as shown in Eq. (1), therefore Bi_2_Te_3_/MASnI_3_ interface shows a relatively low interfacial thermal conductance.

Moreover, it was as low as the experimental values for inorganic/organic interfaces such as a graphene-Bi_2_Te_3_ heterostructure (~ 3.46 MW/(m^2^·K))^59^ and PEDOT:PSS-Bi_2_Te_3_ heterostructure (~ 10 MW/(m^2^·K))^60^ Therefore, these results suggest that the Bi_2_Te_3_/MASnI_3_ interface has a low interfacial thermal conductance, and we expect that the application of this interface to thermoelectric materials can reduce the thermal conductivity. An extremely low thermal conductance is expected even for a stable structure at the Bi_2_Te_3_/MASnI_3_ interface, although the morphological effects are not included in the DMM model. Direct numerical simulations, such as molecular dynamics, may be necessary for further discussion.

### Effective thermal conductivity of the Bi_2_Te_3_ and MASnI_3_ hybrid material

**Figure 10:**
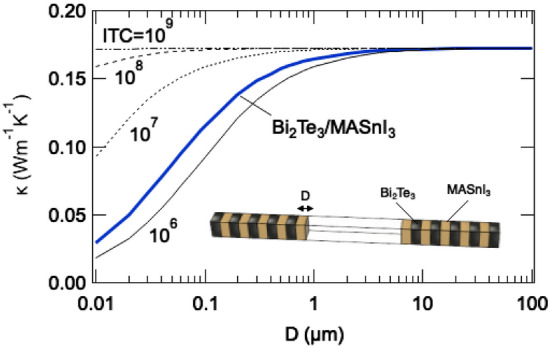
Thermal conductivity of the Bi_2_Te_3_ and MASnI_3_ hybrid material.

Although the actual thermal transport mechanism, such as superlattices with very short periodicity^61^, is too complex to explore here, we have aimed to discuss the effect of the interfacial thermal conductance of Bi_2_Te_3_/MASnI_3_ on the effective thermal conductivity, κ using a simple composite model. Here, a one-dimensional model is used, in which Bi_2_Te_3_ layers and MASnI_3_ layers with a thickness of D µm are alternately arranged, as shown in the inset of Fig. 10; the parameters are the interfacial thermal conductance, ITC, and the thickness of each layer. The results indicate that the effective thermal conductivity of Bi_2_Te_3_/MASnI_3_ asymptotically approaches 0.17 W/(m·K) at a film thickness sufficiently larger than 1 µm. This value is calculated from the experimental values of Bi_2_Te_3_ and MASnI_3_: 2.11 W/(m·K)^62^ and 0.09 W/(m·K)^41^, respectively. This upper limit does not depend on the interfacial thermal conductance because the influence of the interface is negligible in the limit of large D. In contrast, when the film thickness is sufficiently smaller than 1 µm, the interfacial thermal conductance significantly influences the effective thermal conductivity. The blue line in the figure shows the effective thermal conductivity estimated from the calculated interfacial thermal conductance of 1.75 MW/(m^2^·K). The smaller the film thickness, the more effectively the interfacial thermal conductance of Bi_2_Te_3_/MASnI_3_ can be utilized. Thus, it is expected that the thermal conductivity of the Bi_2_Te_3_/MASnI_3_ composite, which consists of small Bi_2_Te_3_ grains in MASnI_3_, will be significantly reduced.

## Conclusion

In this study, we evaluated the stability and electronic state of interface structures of Bi_2_Te_3_ (001) and MASnI_3_ (001), and the thermal conductance of the interface between Bi_2_Te_3_ and MASnI_3_ along the (001) direction was estimated. In the structural optimization, the termination of MASnI_3_ was fixed with SnI_2_ at the interface and surface, whereas for the structure of Bi_2_Te_3_ in contact with MASnI_3_, three termination structures were considered: Te^I^, Te^II^, and Bi termination. After structural optimization, around the Fermi energy, the interface structures with Te^II^ and Bi termination resulted in the formation of interface levels attributed to the overlapping electron densities for both Bi_2_Te_3_ and MASnI_3_ at the interface. It is believed that the formation of interface levels enhances the affinity for the interface structure of Bi_2_Te_3_ and MASnI_3_, and the binding energies for these interface structures are negative. Based on the calculation of the interfacial thermal conductance using DMM, it is expected that the Bi_2_Te_3_/MASnI_3_ interface can significantly reduce the thermal conductivity. These results indicate that the Bi_2_Te_3_/MASnI_3_ composite material is a possible candidate for an excellent thermoelectric material because it has the potential to decrease the thermal conductivity.

## Supplementary Information

Supplementary Information.
