# Supplementary material for: Electronic structure and thermal conductance of the MASnI3/Bi2Te3 interface: a first-principles study
Source: Sci Rep. 2022 Jan 7;12:217. doi: 10.1038/s41598-021-04234-3 (PMC8742053; doi:10.1038/s41598-021-04234-3)
Supplement: Supplementary file 1 — Supplementary Information. [file 41598_2021_4234_MOESM1_ESM.docx]

Supporting Information

Electronic structure and thermal conductance of the MASnI_3_/Bi_2_Te_3_ interface: a first-principles study

Masayuki Morimoto^a^, Shoya Kawano^a^, Shotaro Miyamoto^b^, Koji Miyazaki^b^, Shuzi Hayase^c^ and Satoshi Iikubo^a,d,^*

*^a^Department of Life and Systems Engineering, Kyushu Institute of Technology, Kitakyushu Science and Research Park, Fukuoka 808-0196, Japan*

*^b^Department of Mechanical and Control Engineering, Kyushu Institute of Technology, 1-1 Sensui-cho, Tobata-ku, Kitakyushu 804-8550, Japan.*

*^c^Info-Powered Energy System Research Center (i-PERC), The University of Electro-Communications, 1-5-1 Chofugaoka, Chofu, Tokyo, 182-8585, Japan*

*^d^Department of Advanced Materials Science and Engineering, Faculty of Engineering Sciences, Kyushu University, Kasuga, Fukuoka, 816-8580, Japan*

*Corresponding Author. E-mail addresses: iikubo.satoshi.472@m.kyushu-u.ac.jp


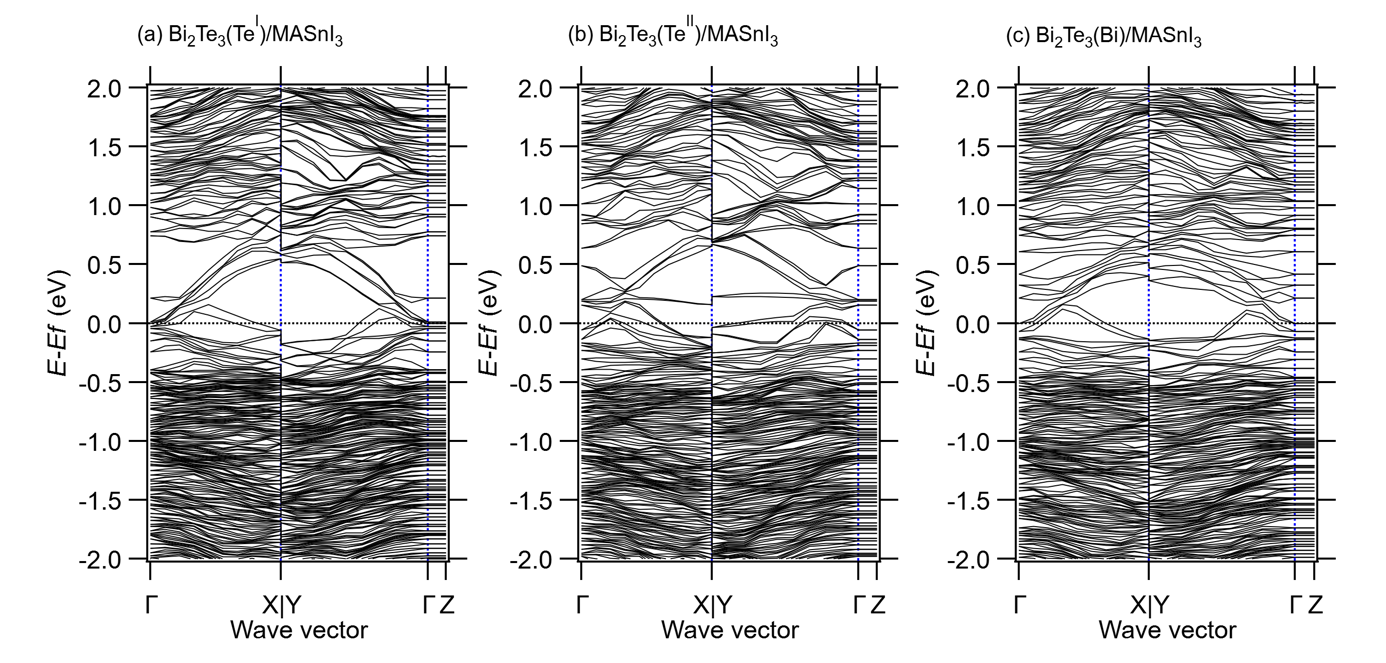


Figure S1. Band structures of (a) Bi_2_Te_3_(Te^I^)/MASnI_3_, (b) Bi_2_Te_3_(Te^II^)/MASnI_3_, and (c) Bi_2_Te_3_(Bi)/MASnI_3_ interface structure.
